# Perceptions of hookah smoking harmfulness: predictors and characteristics among current hookah users

**DOI:** 10.1186/1617-9625-5-16

**Published:** 2009-12-18

**Authors:** Khaled Aljarrah, Zaid Q Ababneh, Wael K Al-Delaimy

**Affiliations:** 1 Applied Physics Department, Jordan University of Science and Technology, Irbid, Jordan; 2 Physics Department, Yarmouk University, Irbid, Jordan; 3 Family & Preventive Medicine Department, University of California San Diego, 3855 Health Sciences Dr, La Jolla, CA, 92093, USA

## Abstract

**Introduction:**

Tobacco cigarette smoking a well-known cause of cancer and other diseases. Hookah smoking is another form of tobacco use that has rapidly spread in the United State and Europe. This study assessed beliefs about the harmfulness of smoking hookah.

**Methods:**

We surveyed hookah users in all cafes that provided hookah to its customers in downtown San Diego, California and nearby areas. A total of 235 hookah users participated in this study.

**Results:**

Average age of study participants was 22 years, 57% were males, and 72% were not cigarette smokers. Whites were more likely to use hookah than the other ethnic groups (33%), older hookah users (26-35 years) were mostly males, and mint flavor of hookah tobacco was the most popular among a wide variety of flavors (23%). There was no significant difference in gender in relation to the wrong perception that hookah is less harmful than cigarettes, but those of Asian ethnicity were much less likely than other ethnic groups to believe that hookah is less harmful than cigarettes. More frequent users of hookah were more likely to believe that hookah is less harmful than cigarettes. The majority of hookah users (58.3%) believe hookah is less harmful than cigarette smoking.

**Discussion:**

Compared to cigarettes, there appears to be a lack of knowledge about the harmfulness of smoking hookah among users regardless of their demographic background. Education about the harmfulness of smoking hookah and policies to limit its use should be implemented to prevent the spread of this new form of tobacco use.

## Introduction

Lung cancer is the leading cause of cancer mortality in the world [1]. Cigarette smoking cause up to 87% of lung cancer deaths worldwide and is estimated to cause up to 90% of lung cancers in the United States [2-4]. Smoking is also responsible for cancers of larynx, oral cavity, pharynx, esophagus, and bladder [4,5]. Moreover, continued smoking increases resistance to cancer therapy in lung cancer patients [6,7]. The smoke from tobacco contains more than 60 carcinogens of both radioisotopes and chemicals [8] that in additional to cancer can cause several respiratory ailments and increase in respiratory infections. Nicotine contained in cigarette smoke causes a depression in the immune response to malignant growths in exposed tissue [9].

Hookah smoking is another form of tobacco use. Hookahs originated in India in the 15^th ^century and then spread to the Near East countries. Hookahs spread first to Persia and underwent further changes to its original shape to the current known shape. In the middle of the 16^th ^century, hookahs reached the Ottoman Empire, Egypt, and other Mediterranean regions [10]. Late in the 19^th ^century, hookah use started spreading among women in the Middle East [11,12]. In the last two decades, the use of hookah has been gradually increasing in Europe and the USA [13-16]. Hookahs are known around the world by many different names and slightly different forms: water pipe, hubble-bubble, nargeela, nargileh, argeela, arghileh, shisha, sheesha, okka, kalian, ghelyoon, ghalyan, boury, and gouza. Originally, the tobacco smoked in hookahs did not have any additives, but lately, the "Ma'ssell" was introduced, which is a mixture of tobacco, molasses, and often a flavor or fruit extract. Adding fruit to hookah base provides more flavors. The smell of the smoke-flavored tobacco, social ambiance with friends and family, and easy access to hookahs are some of the factors attributed to its dramatic spread. Different methods of providing hookahs in cafe lounges with coffee or other drinks and the ability of the user to handle the charcoals for the hookah gives the practice a special culture that is probably helping hookah spread and popularity [17].

Some perceive hookah is not harmful [18-21] because of the belief that the smoke gets filtered in the water [22,23], but it is not clear if this perception is widespread or different according to demographic and population characteristics. Scientific facts indicate that when compared to cigarette smoking, the number of puffs and volume from using hookahs are about ten times higher than cigarettes [19,24]. Hookah smoke also contains 36 times the amount of nicotine and higher concentrations of heavy metals [24,25]. The burning temperature of tobacco for hookah use is about 900°, compared to 450° for cigarettes, which could produce different type and levels of harmful chemicals and tar [24]. Further, exhaled CO levels from hookah users were twice as high as cigarette smokers in cessation programs [21]. Like cigarettes, hookah use is also a health hazard to non-smokers because of secondhand smoke and it can lead to the transmission of infectious diseases, since the same hookah mouthpiece can be used by many people during the same smoking session [26-28]. Hookah use has been shown to cause an acute increase in heart rate and systolic and diastolic blood pressure [29]. Studies have shown that using the water as a filter in the hookah did not change the level of nicotine in the smoke compared to that without using the water [24,30].

Data on the perception of risk of hookah use compared to that of cigarettes are limited and vary from one region to another. Because of current rapid increases in hookah use in the US, more data are needed about those who use it and their perceptions. Previous studies about hookah use in the United States involved Internet surveys, university students [20,31,32] or the military [18]. Two studies specifically surveyed hookah users in the US. One study was limited to a single cafe in Richmond, Virginia and a sample from an Internet hookah forum [17]. Participants in the second study were recruited via flyers that were distributed in two cities (Richmond, VA and Memphis, TN), and participants were interviewed in a lab setting [33]. Participants in both studies were paid volunteers. In this study, a survey of hookah users from hookah cafes in San Diego, California was performed to assess characteristics and perceptions of users in the general population in Southern California in relation to the believe about hookah smoking harmfulness. Knowing the characteristics of the hookah users according to their belief about harmfulness of smoke from hookah tobacco would help develop health promotion initiatives and interventions that specifically address the sub-population of users who need it most.

## Methods

An Internet search was made for any venue providing hookah smoking within San Diego. These were mapped and all the venues of restaurants, cafes, and clubs located geographically in downtown and up to 15 miles in any direction were included. Venues that did not match these criteria were contacted and asked about serving hookah smoking and places that only sold hookah paraphernalia and related tobacco products were excluded. Participants were those who were smoking hookahs in these venues at the time of data collection. Data were collected from nearly all cafes that offered hookahs lounge in downtown San Diego, California and the surrounding suburb area.

Data was collected during a four-week period in August and September 2008 during the weekdays and on weekends. Most collection occurred on Friday and Saturday nights because of the large number of hookah smokers available during these periods. Owners of the 10 venues selected for this study were initially approached to obtain their permission and all of the owners agreed to allow the survey to be conducted. After obtaining verbal approval from the hookah users, we then asked them to sign a consent form and fill in the questionnaire. Similarly, there was a very high response rate among hookah users approached for the survey; only one person refused to participate. All participants sitting in the cafes were given the surveys unless they said they did not smoke hookah. A total of 256 persons participated in this study; 21 people from this group were excluded because they stated in the survey that they were non-hookah users. Some of them requested to complete the survey even though they did not smoke hookah.

### Questionnaires

The survey was brief, since longer surveys can disrupt a business and lead to a poor response rate from customers who want to enjoy their time. Questions were included about the participant's gender, ethnicity, and age. In order to assess the association between cigarette and hookah smoking, participants were asked about cigarette smoking, number of cigarettes smoked a day and brand of cigarettes. Hookah users were asked for their favorite hookah tobacco flavor, and how often they smoked hookah. The final question assessed their opinion about the harmfulness of hookah smoking compared to cigarette smoking.

### Data analysis

Descriptive analyses of means, medians and percentages were calculated for the variables in this study. Data from the questionnaire were categorized for the purpose of analyses. We also carried out analyses by gender. The different cigarette brand and hookah flavor were assigned as "Other" if only 3 or less participants reported them. Age was dichotomized at the median of 25 years. A chi-square test was carried out on categorized variables to determine the level of significance between the different variables of the study.

We initially assessed the harmfulness perception of smoking hookah according to characteristics of our study population and determined significance using a chi-square test. Any participant who did not answer the question about harmfulness was not included in the final analyses.

## Results

Details of participant demographics and their beliefs about hookah use are described in Table 1. Out of 235 participants, 57% were males. White participants represented 32.9% of the study subjects, followed by Latino (22.4%), Middle Eastern (21.5%), Asian (11.0%), and African American (6.6%) ethnicities. The average age of participant was 21.8 years. Among all participants, only 28.4% were cigarettes smokers, with a median number of cigarettes smoked per day of 6 cigarettes. Most of the cigarette smokers smoked the Camel brand. A total of 35.2% of hookah users smoke every week and a smaller percentage (27%) smoke every 6 months, every month (24.4%) and every day (13.5%).

**Table 1:** Characteristics of study participants (n = 235).

Characteristics	N	% of Participants
**Gender**		
Male	134	57
Female	101	43
**Age **(avg) [range: 17-35]	172	21.8
**Ethnicity**		
White	75	32.9
African American	15	6.6
Asian	25	11
Latino	51	22.4
ME	49	21.5
Other	13	5.7
**Hookah use**		
Every day	31	13.5
Every week	81	35.2
Every month	56	24.4
Every six month	62	27
**Smokes cigarettes**		
Yes	67	28.4
No	168	71.6
**Cigarette brand**		
Marlboro	18	30
Camel	30	50
Other	12	20
**Number of cigarette a day **(median, range)	6, 1-27	

**Figure 1:**
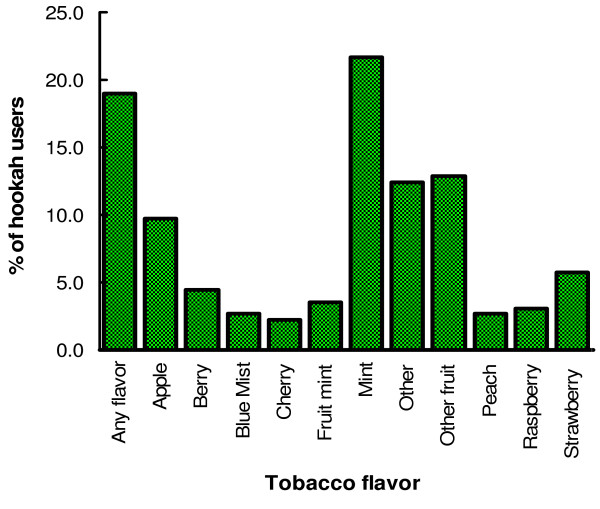
**Frequencies of reported favorite tobacco flavor in hookahs among users in San Diego**.

Frequencies of reported favorite hookah flavors are shown in Figure 1. Mint flavor seems to be the most common-used flavor and was chosen by 22% of our study subjects. Individual fruit flavors as the favorite flavor for individuals were mostly less than 5%, while a larger percentage of participants (19%) preferred a combination of fruit flavors.

Characteristics of participants by gender are described in Table 2. There was no significant difference in the ethnicity according to gender. Whites were more frequent users of hookah, followed by Middle Eastern and Latino participants in both genders. Most males and females were non-cigarette smokers. Age was different between males and females, where more females were in the younger age group than males (p = 0.02). Females were less likely than males to smoke hookah every day and more likely to smoke every 6 months, although this did not reach statistical significance.

**Table 2:** Male and Female demographic, characteristics, tobacco use, and beliefs about hookah smoking harmfulness among hookah users in San Diego.

Variable	Category	Male		Female		P-Value
		N	%	N	%	
**Ethnicity**	White African American Asian Latino ME Other	39 10 15 28 29 10	29.8 7.6 11.5 21.4 22.1 7.6	35 6 9 23 22 4	35.42 6.0 9.1 23.2 22.2 4.0	0.80
						
**Smoke cigarettes**	Yes No	37 94	28.2 71.8	27 74	26.7 73.3	0.80
						
**Age**	17 - 25 26 - 35	76 20	79.2 20.8	69 6	92 8	0.02
						
**Hookah use**	Every day Every week Every month Every 6 months	24 45 32 31	18.2 34.1 24.2 23.5	7 36 24 31	7.1 36.7 24.5 31.6	0.09
						
**Hookah harm**	Less	82	62.6	52	52.5	
**compared to cigarettes**	More	13	9.9	13	13.1	0.31
	Similar	36	27.5	34	34.3	

A majority of hookah users (58.3%) believe hookahs are less harmful than cigarettes and only 30.4% believe they are more harmful than cigarettes. Belief about harmfulness of smoking hookahs compared to cigarettes was very similar between males and females. Participants' beliefs about the risk associated with hookah smoking compared to cigarettes are shown according to their characteristics in Table 3. There was no significant difference in belief of harmfulness of smoking hookah according to smoking status (p = 0.58), frequency of hookah use (p = 0.55), or age (p = 0.84). Only ethnic groups were statistically significant in their perception and belief about the harmfulness of hookah smoking. Whites, African Americans and Middle Eastern ethnic groups were much more likely than Asians and slightly more than Latinos to believe hookah is less harmful than cigarettes (p = 0.03).

**Table 3:** Hookah user belief on the harmfulness of hookah smoking by characteristics.

Variable	Category	Less		Similar		More		P-Value
		n	%	n	%	n	%	
Smoke cigarettes	Yes No	33 99	53.2 60.0	7 19	11.3 11.5	22 47	35.5 28.5	0.58
								
Hookah use	Every day Every week Every month Every 6 months	21 47 31 31	70.0 59.5 56.4 50.8	1 10 5 9	3.3 12.7 9.1 14.8	8 22 19 21	26.7 27.8 34.5 34.4	0.55
								
Ethnicity	White African American Asian Latino ME Other	47 11 9 28 33 5	64.4 68.8 37.5 57.1 64.7 38.5	4 1 3 8 9 0	5.5 6.3 12.5 16.3 17.6 0.0	22 4 12 13 9 8	30.1 25.0 50.0 26.5 17.6 61.5	0.03
								
Age	17 - 25 26 - 35	82 15	57.7 57.7	16 2	11.3 7.7	44 9	31.0 34.6	0.84

Using logistic multivariate regression analyses to predict the belief that hookah is less harmful vs. same as or more harmful than cigarettes, none of the variables were significantly related to such belief when put in the model (data not shown).

## Discussion

Our study suggests there is a widespread perception among hookah users that it is less harmful than cigarette smoking and it is independent of gender, ethnicity, age or smoking status of users. Our population-based study of hookah-bar patrons in San Diego included all hookah bars in the downtown area and surveyed patrons from different ethnic groups and both genders. Hookah users were mostly young men and women below the age of 25 years.

The percentage of smokers in our study population of hookah users was 28.4%, which is higher than the 13-16% smoking prevalence in the general California population for those aged 18-44 years [34]. However, smoking prevalence among hookah users in our study was very comparable to that in the general California population as represented by the California Tobacco Survey; which is a State-wide survey of a representative population from California that found that 30.6% of hookah users were current smokers in 2008, which is the same year the current study was conducted. Therefore, our study sample of hookah users resembles the larger California population in terms of smoking prevalence.

It therefore appears that hookah smokers are more likely to be cigarettes smokers. This was also found in other studies from US cities, although the cigarette smoking rates for hookah users varied widely, from 63% in Richmond, VA [33] to 58% in Pittsburg, PA [20] and 35% in Memphis, TN [33]. This may reflect the difference in sampling from web-based and volunteer study subjects to random university sample compared to hookah café users in our study. This could also be explained by the difference in the population of hookah users between regions in the US. This difference is important to document and understand for the purpose of determining future hookah control programs and initiatives.

A study from the United Kingdom found cigarette smoking was the most important predictor among those who ever tried hookahs to become regular hookah users [21]. In our study, smoking cigarettes did not significantly influence the belief that hookah is less harmful than cigarettes; a majority of hookah users believed it was less harmful than cigarette smoking. Recent smaller studies from different US populations confirm this misperception [17,20,31-33]. A qualitative analyses of attitudes among 12 hookah users in the UK and Canada also support the perception among users that hookah use is less harmful than cigarettes [35].

Perception that the hookah smoke is filtered in the water seems to be one the main beliefs justifying the less harmful influence of hookahs [22,23]. However, it is well known that passing air bubbles through water does not change their contents, and since the volatile carcinogens for tobacco smoke and other particles will stay within the air bubble during its passage through the water, the water will not filter the smoke in the bubbles. Some hookah users report hookah smoke being less irritating than cigarette smoking, noting it has a 'smooth texture' that allows them to smoke it for hours [35]. More importantly, the negative social norm against cigarette smoking is not applied to hookah because of its more recent trend and use. This may be contributing to the wide and dramatic spread of this type of tobacco use.

It is a public health concern that non-cigarette smokers believe that hookahs are less harmful than cigarette use because those who did not smoke until becoming adults passed the period of adolescence and early adulthood when they are most vulnerable to cigarette smoking. This group may be reintroduced to the habit of cigarette smoking through hookah use or continue to be regular hookah users and get exposed to the harms of tobacco use. In conducting this study, there were several friends of hookah users who were sitting at the same table in the hookah café but reported never smoking a hookah. Given that previous studies show most of the hookah users started with friends in café restaurants [17,33] and since this has mostly become a group socializing activity, we believe those nonsmokers will eventually try a hookah and become users along with their friends.

Higher frequency of hookah use was positively related to the belief that it is less harmful than cigarettes; 70% of every-day hookah users believe it is less harmful than cigarettes while only 50% of those who use it every six months believe it is less harmful than cigarettes. This did not reach statistical significance. This suggests that there is a risk that irregular users will gradually become regular more frequent users based on their belief this will not be as harmful to their health.

Our ethnic distribution of Latinos, Asians and African Americans were demographically comparable to that for California, but there were less Whites and a much higher Middle Eastern ethnic group in our study sample. Middle Eastern ethnicity is usually categorized as White in the general census. The much higher use by the Middle Eastern ethnic group was also found in a study in the UK, where they were twice as likely to use hookah than other ethnic groups [21].

Another observation we noted during data collection was a tendency for similar ethnicities to frequent the same hookah bars. This is part of the appeal of hookah use, as a social group activity for people with similar backgrounds. In addition, the exotic relaxed atmosphere, the nice sweetened scents from the flavored hookah tobacco smoke, and the relatively cheap costs of smoking a hookah contribute to its use among mostly young adults [26]. Asians in our sample were significantly more likely to believe a hookah is more harmful than cigarettes. However, this was not significant in the multivariate analyses and may be due to the small number of subjects in this group. Further exploration of this finding is needed from future studies. Other comparable studies in the US mostly included White ethnic groups.

There was no influence of gender on the perceptions and use of hookah from our study. The hookah originated in Asia and its use for many decades in recent history was dominated by males. However, re-birth of this habit in modern age among young adults in the Middle East is spreading among females [11,16,26,36,37] due to social acceptability even in traditionally conservative societies like Saudi Arabia. Previous studies in the US show a large variation in participation of hookah users according to gender [17,20,33].

Mint flavor is the highest single hookah tobacco flavor preferred by users in our study. This flavor has also been popular in the US by smokeless tobacco and cigarette users Outside the US, it is not known which flavors predominate. The numerous varieties of flavors and the fact the most users used or preferred all flavors suggest a risk for continued use and exploration by users of the different flavors of tobacco.

Our study is one of the larger studies in the United States that addresses characteristics of hookah users by targeting them in the general population as café patrons. Previous studies recruited volunteers who were provided incentives for volunteering. Although our study is not a random representative sample of hookah users, the fact that all major hookah cafes were included gives some confidence about the representativeness of such users. Some San Diego cafes that do not have a web address or are not listed on the Internet might have been missed, but this is unlikely, since the majority of businesses are on the Internet and list web sites for the hookah bars. We are not able to comment on users who exclusively smoke at home; those users may be underage teens. The short questionnaire prevented in-depth exploration of attitudes and behaviors about hookah use. However, this was not a qualitative analyses and the aim was to assess the characteristics and perceptions of hookah café patrons.

In conclusion, we found a concerning trend of emerging use of hookah and the belief that it is less harmful than cigarette use. Most of the hookah users were non-cigarette smokers. Both current smokers and nonsmokers had comparable views and therefore there is a risk that this will become a new tobacco use trend for never smokers. The exotic, social, and group nature of this habit is appealing to young adults, regardless of gender or ethnicity. Middle Eastern ethnicity seems to be the most vulnerable group for hookah use. Culturally-targeted public health campaigns to educate and disseminate to the younger population about the harmful effects of hookah are urgently needed. Health policy initiatives should be formulated to prevent marketing and licensing of hookah tobacco products and paraphernalia in local markets and shops. Further studies on the spread of hookah use among underage teens who are unlikely to frequent the hookah bars are needed. We also believe future studies should directly quantify the harmfulness of hookah smoking by determining pulmonary and other vital functions among users.

## Competing interests

The authors declare that they have no competing interests.

## Authors' contributions

KJ helped design the study, carried out data collection, data analysis, and drafted the manuscript. ZQA carried out the statistical analysis and interpretation. WKA conceived the study, supervised the data collection and analyses, and helped draft the manuscript. All authors read and approved the final manuscript.
